# Human Tyrosinase Displayed on the Surface of Chinese Hamster Ovary Cells for Ligand Fishing of Tyrosinase Inhibitors from Medicinal Plants

**DOI:** 10.3390/molecules30010030

**Published:** 2024-12-25

**Authors:** Xiao-Rui Zhai, Ming-Jie Li, Xiang Yin, Ayzohra Ablat, Yuan Wang, Peng Shu, Xun Liao

**Affiliations:** 1 Chengdu Institute of Biology, Chinese Academy of Sciences, Chengdu 610041, China; zhaixr@cib.ac.cn (X.-R.Z.); yinxing@cib.ac.cn (X.Y.); ayi@cib.ac.cn (A.A.); 2 University of Chinese Academy of Sciences, Beijing 100049, China; 3 HBN Research Institute and Biological Laboratory, Shenzhen Hujia Technology Co., Ltd., Shenzhen 518000, China; limingjie@hbn.cn (M.-J.L.); wangyuan@hbn.cn (Y.W.)

**Keywords:** cell surface display, Chinese hamster ovary cells, tyrosinase inhibitors, *Alhagi sparsifolia*, *Coffea arabica*, B16 melanoma cells

## Abstract

Ligand fishing is a promising strategy for the screening of active ingredients from complex natural products. In this work, human tyrosinase (hTYR) was displayed on the surface of Chinese hamster ovary (CHO) cells for the first time; it was then used as bait to develop a new method for ligand fishing. The localization of hTYR on the CHO cell surface was verified by an enzyme activity test and fluorescence microscopy. The displayed tyrosinase (CHO@hTYR) maintained relatively stable enzymatic activity (82.59 ± 2.70%) within 7 days. Furthermore, it can be reused for fishing five times. Guided by the proposed ligand fishing method, four tyrosinase inhibitors, including 4-methoxy-5-methyl coumarin (**1**), cupressuflavone (**2**), amentoflavone (**3**), and 3,4-dimethoxy-5-methyl coumarin (**4**), were isolated from *Alhagi sparsifolia*, and the active fraction with low polarity was isolated from *Coffea arabica*; these two medicinal plants possess skin-lightening potential. All the isolated tyrosinase inhibitors significantly reduced the intracellular tyrosinase activity and melanin level in B16 cells enhanced by α-MSH. Meanwhile, the active fraction (100 μg/mL) from *C. arabica* exhibited stronger inhibitory effects than the positive controls (α-arbutin and kojic acid) by recovering them to the normal levels. This work demonstrated the promising application of the cell surface display in the field of ligand fishing and is helpful in unveiling the chemical basis of the skin-lightening effect of *A. sparsifolia* and *C. arabica*.

## 1. Introduction

Tyrosinase is a copper ion-containing polyphenol oxidase which is prevalent in animals, plants, and microorganisms. As a key rate-limiting enzyme in melanin synthesis, tyrosinase influences the pigmentation of human skin, hair, and eyes [1]. The increased tyrosinase activity may result in hyperpigmentation of the skin or even malignant melanoma [2,3]. Furthermore, a high level of tyrosinase may accelerate the production of neuromelanin and induce neurodegenerative diseases such as Parkinson’s disease [4]. Therefore, it is of great significance to discover safe and effective tyrosinase inhibitors. Medicinal plants represent a rich source of natural compounds, from which a lot of tyrosinase inhibitors have been isolated. However, to screen active compounds from a complex mixture of plant extracts is always challenging. To date, various affinity-based methods have been developed for this purpose, including ligand fishing [5,6], affinity ultrafiltration [7], and cell membrane affinity chromatography [8], among which ligand fishing based on immobilization of the target protein has drawn increasing attention due to its high specificity and ease of operation. Recently, we reported a new strategy for ligand fishing in which the target protein was displayed at the surface of *E. coli* by molecular engineering instead of being immobilized on solid materials via covalent binding, providing a “natural” conformation of the protein to ensure a more reliable screening [9,10]. Currently, the screening of tyrosinase inhibitors relies heavily on the cheap mushroom tyrosinase in both the enzymatic assay and ligand fishing [11]. But since significant differences exist between the tyrosinases that originate from mushrooms and those from humans, as far as the protein structure and function are concerned [12], it is not surprising that a large amount of the screening based on mushroom tyrosinase offers false results [13]. Therefore, we propose a ligand fishing method based on a cell surface display of human tyrosinase to provide a much more reliable and efficient strategy for the screening of the enzyme inhibitors.

Chinese hamster ovary (CHO) cells are the preferred host for the production of recombinant proteins in the biopharmaceutical industry. These mammal-derived cells can contribute to proper folding and secretion of the recombinant proteins, allowing them to be much closer to their natural counterparts [14]. For example, monoclonal antibodies, interferons, and other therapeutic agents produced by CHO cells were used for the treatment of cancer and various diseases [15]. Furthermore, CHO recombinant cells can be used as whole-cell catalysts to improve the catalytic efficiency [16]. The excellent proliferation and protein expression capabilities of CHO cells make them an optimal choice for the expression of human tyrosinase.

*Alhagi sparsifolia* Shap., which belongs to the family of Fabaceae, is distributed in Central and East Asia, mainly in Inner Mongolia, Gansu and Qinghai provinces, and the Xinjiang Uyghur Autonomous Region in China [17]. It is a representative species in arid and semiarid regions. Because it is the major food for camels in the extremely arid region, it is called camel thorn in China. *A. sparsifolia* has been utilized in traditional Uyghur medicine in the treatment of various ailments, including fever, inflammations, headache, toothache, hypertension, hyperlipidemia, neuroprotection, and liver disorders [18,19,20,21]. Chemical compounds isolated from this plant have been reported to possess antioxidative, antinociceptive, anti-inflammatory, and antibacterial activities [22,23]. Recently, methanol extracts of this plant were found to inhibit the activity of tyrosinase, indicating its potential for skin-whitening purposes [24]. *Coffea arabica* L., which belongs to the Rubiaceae family, is one of the most popular drinks worldwide. *C. arabica* possesses various health benefits that prevent cardiovascular disease, type 2 diabetes, Parkinson’s disease, and Alzheimer’s disease [25]. Active compounds in *C. arabica*, such as gamma-tocopherol, may help prevent skin pigmentation [26]. Additionally, it is reported that the two key ingredients of *C. arabica*, i.e., caffeic acid and ferulic acid, work synergistically to scavenge free radicals of the skin and mitigate oxidative damage caused by ultraviolet rays [27,28]. While previous studies have indicated the skin-whitening effect of these two plants, the active ingredients that inhibited tyrosinase have not been identified.

In this study, we displayed human tyrosinase on the surface of CHO cells and tested the storage stability and reusability of the recombinant cells. Using the recombinant cells as bait for ligand fishing, a new method for screening tyrosinase inhibitors was developed and optimized. Finally, the inhibitors present in *A. sparsifolia* and *C. arabica* were screened by this method, and their chemical structures were identified by NMR, HPLC-MS, or GC-MS techniques. Their effects on the intracellular activity of tyrosinase and the melanin content were assayed in B16 melanoma cells.

## 2. Results and Discussion

### 2.1. Localization of Human Tyrosinase on CHO Cells

**Figure 1:**
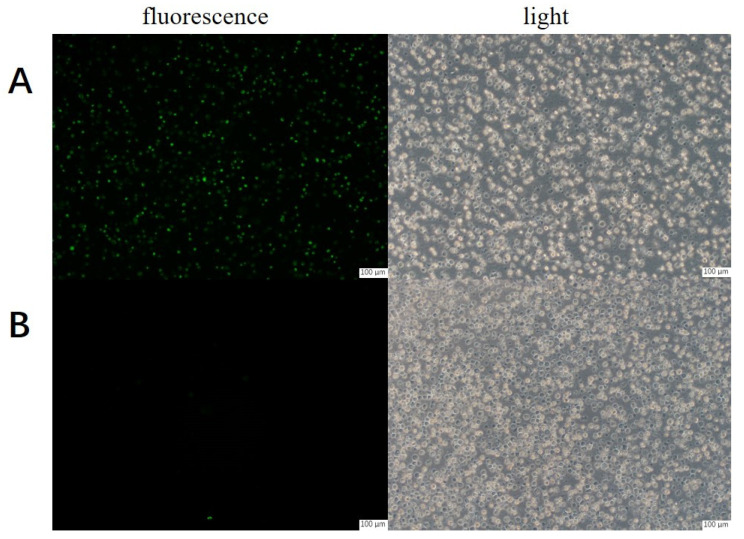
Fluorescence microscope images of CHO@hTYR and control cells. (**A**) CHO@hTYR; (**B**) control cells; (**left**): fluorescence; (**right**): light; scale bars: 100 μm.

The *homo tyrosinase* gene was successfully inserted into the pcDNA3.1 (+) vector (Supplementary Data, Part S1) to obtain the recombinant vector (pCDNA3.1/hTYR), which was stably transfected into the CHO cells. At the same time, the green fluorescent protein (GFP) expressed by the recombinant CHO was used to monitor the transfection efficiency of the hTYR, as illustrated in Figure 1. In contrast, control cells that were transfected with pcDNA3.1 (+) displayed no fluorescence, confirming the successful construction of CHO@hTYR.

### 2.2. Enzymatic Activity and Stability of CHO@hTYR

**Figure 2:**
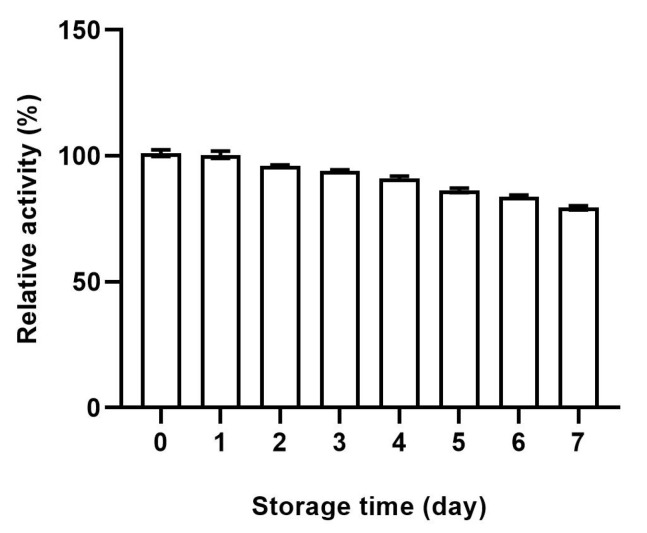
Storage stability of CHO@hTYR cells.

The tyrosinase activity of the recombinant cells was tested to verify the activity of the displayed enzyme. The enzyme activity of the recombinant cells was 99.32 ± 4.66 U/1 × 10^4^ cells relative to the mushroom tyrosinase. The calculation method is detailed in the Supplementary Data, Part S2. The control cells transfected with empty vector exhibited no ability to catalyze L-DOPA. For the stability of the recombinant cells, we monitored the enzymatic activity in a consecutive period of 7 days. As shown in Figure 2, the enzymatic activity slowly decreased, and it retained more than 80% of the initial recombinant cells of the 0 day, suggesting the good storage stability of the recombinant cells.

### 2.3. Optimization of the Conditions for Ligand Fishing

**Figure 3:**
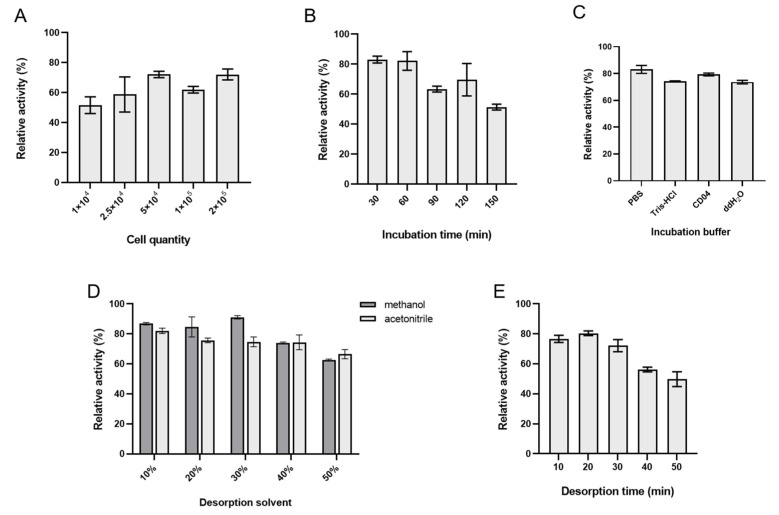
Optimization of the conditions for ligand fishing: (**A**) cell quantity; (**B**) incubation time; (**C**) incubation buffer; (**D**) desorption solvent; (**E**) desorption time. Data are presented as mean ± SD. The experiments were performed in triplicate.

The conditions for ligand fishing were optimized based on five parameters, including cell number, incubation time, incubation buffer, desorption time, and the desorption solvent. Using 1 mL of quercitrin (0.1 mM) as the hTYR ligand, the remaining enzyme activity of CHO@hTYR after each round of fishing was assessed to reflect its robustness as the bait. The enzyme activity was tested as described in Section 3.4. It was observed that the enzymatic activity of the recombinant cells no longer increased when the number of cells reached 5 × 10^4^, suggesting that this cell number was the best for the ligand fishing (Figure 3A). As indicated in Figure 3B, 30 min of incubation was enough to bind the ligand, while the remaining enzymatic activity decreased when the incubation time was above 90 min. For the choice of buffer solution, PBS proved to be the most effective one for maintaining the TYR activity of CHO@hTYR (Figure 3C). Thirty percent methanol was chosen as the desorption solvent since it kept the most enzymatic activity of the recombinant cells (Figure 3D). The optimal desorption time was determined to be 20 min, as indicated in Figure 3E. Therefore, the optimal conditions for ligand fishing were determined as follows: a cell volume of 5 × 10^4^, an incubation time of 30 min, PBS as the buffer system, a desorption time of 20 min, and 30% methanol as the desorption solvent. Under these optimized conditions, the limit of detection (LOD) for quercitrin was 0.166 μM (Part S3 in Supplementary Data). Furthermore, the CHO@hTYR can be reused up to five times, as illustrated in the Supplementary Data (Figure S5).

### 2.4. Selectivity of CHO@hTYR in Ligand Fishing

**Figure 4:**
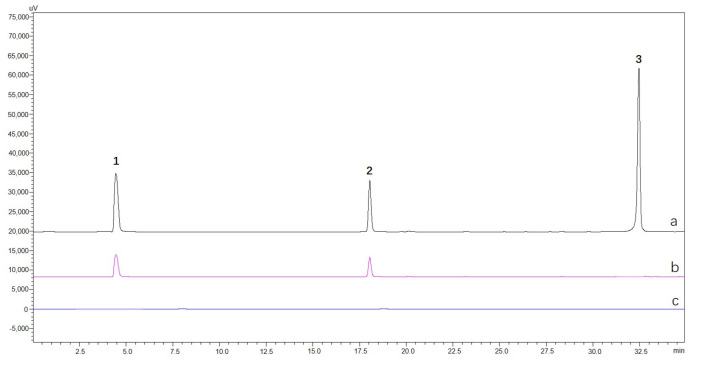
HPLC chromatograms of (a) mS0, (b) mS5, and (c) mS5−blank. Peak identities: **1**, α−arbutin; **2**, quercitrin; **3,** dihydrotanshinone I.

One non-ligand of TYR, dihydrotanshinone I, and two ligands (α-arbutin and quercitrin) were mixed to prepare a standard mixture designated as mS0. The fishing assay was carried out as outlined in Section 3.6, affording the final eluate which was referred to as mS5. The CHO cells were utilized as a blank control to replicate the fishing process above; the control was recorded as mS5-blank. As illustrated in Figure 4, CHO@hTYR cells are able to capture only the two ligands, demonstrating the good selectivity of the proposed method.

### 2.5. Screening and Identification of the hTYR Ligands from Extract of A. sparsifolia and C. arabica

**Figure 5:**
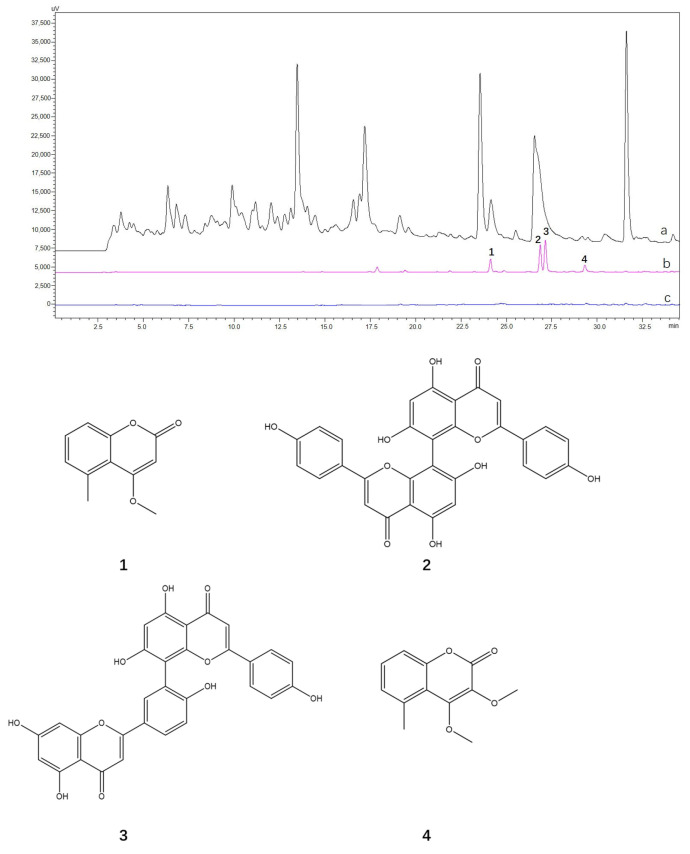
HPLC chromatograms of ligand fishing of *A. sparsifolia*: (a) S0−A, (b) S5−A, and (c) S5−A−blank. Chemical structures of 4−methoxy−5−methyl coumarin (**1**), cupressuflavone (**2**), amentoflavone (**3**), and 3, 4−dimethoxy−5−methyl coumarin (**4**).

**Figure 6:**
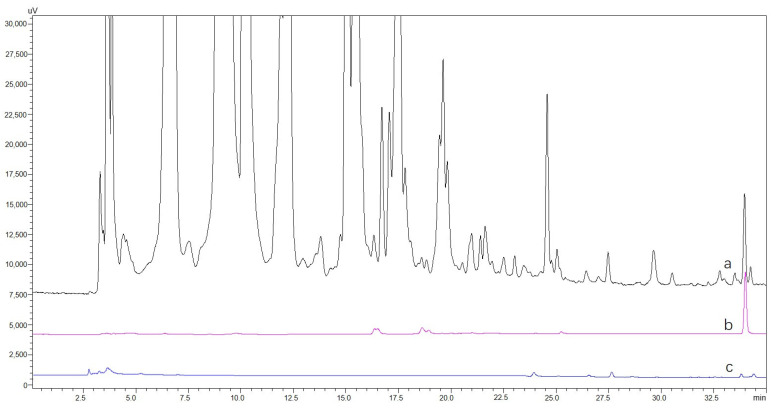
HPLC chromatograms of ligand fishing of *C. arabica*: (a) S0−C, (b) S5−C, and (c) S5−C−blank.

The extraction solutions of *A. sparsifolia* and *C. arabica* were prepared at a concentration of 1 mg/mL (S0-A and S0-C, respectively), and 5 × 10^4^ of CHO@hTYR cells were used to fish the hTYR ligands. The final solutions eluted with 30% methanol were designated as S5-A and S5-C, respectively. The CHO cells were used to fish those two S0s to afford S5-A-blank and S5-C-blank. Four compounds were rapidly screened from *A. sparsifolia*, as illustrated in Figure 5, and their structures were then identified using MS and NMR. As shown in Figure S6, compounds **1**–**4** yielded the pseudo-molecular ions of *m*/*z* 191.11 [M+H]^+^, 539.64 [M+H]^+^, 539.10 [M+H]^+^, and 221.08 [M+H]^+^, respectively. Furthermore, based on the ^1^H and ^13^C NMR data (Supplementary Data, Figure S6 and Table S2), these compounds were identified as 4-methoxy-5-methyl coumarin (**1**), cupressuflavone (**2**), amentoflavone (**3**), and 3,4-dimethoxy-5-methyl coumarin (**4**), respectively [29,30,31,32]. The active compounds in S5-C from the *C. arabica* extract exhibited very low polarity (Figure 6); thus, it was difficult to purify them. Therefore, the chemical compounds were identified by GC-MS according to the GB 5009.168-2016 National Food Safety Standard “Determination of fatty acid in food”, using an internal standard method [33], with an 80–110% recovery rate and ≤3% relative standard deviation (Supplementary Data, Figure S8), the main components of which were linoleic acid (55.18 g/100 g), palmitic acid (26.24 g/100 g), ricinoleic acid (6.58 g/100 g), and stearic acid (5.31 g/100 g).

Preliminary experiments using magnetic nanoparticle-immobilized mushroom tyrosinase (MNP@mTYR) were carried out to screen the enzyme inhibitors from both plants (Supplementary Data, Part S6). While no active compounds were found from *C. arabica*, two active compounds, quercitrin and 8-hydroxyluteolin, were successfully extracted from *A. sparsifolia*; the outcome of this was different from that of the CHO@hTYR-based ligand fishing. This is probably due to the different origin of the enzyme, which may lead to variations in protein structure and active sites, influencing the binding of different ligands. On the other hand, different modes of immobilization of the TYR may also lead to this result. Human tyrosinase is too costly to be used for large-scale enzyme immobilization. In this work, the CHO cells stably transfected with human tyrosinase addressed this issue by providing a sufficient quantity of the enzyme with good stability and reusability, ensuring a more viable result for the screening of inhibitors of the enzyme.

### 2.6. Inhibitory Effects on Tyrosinase Activity and Melanin Content in α-MSH-Induced B16 Melanoma Cells

**Figure 7:**
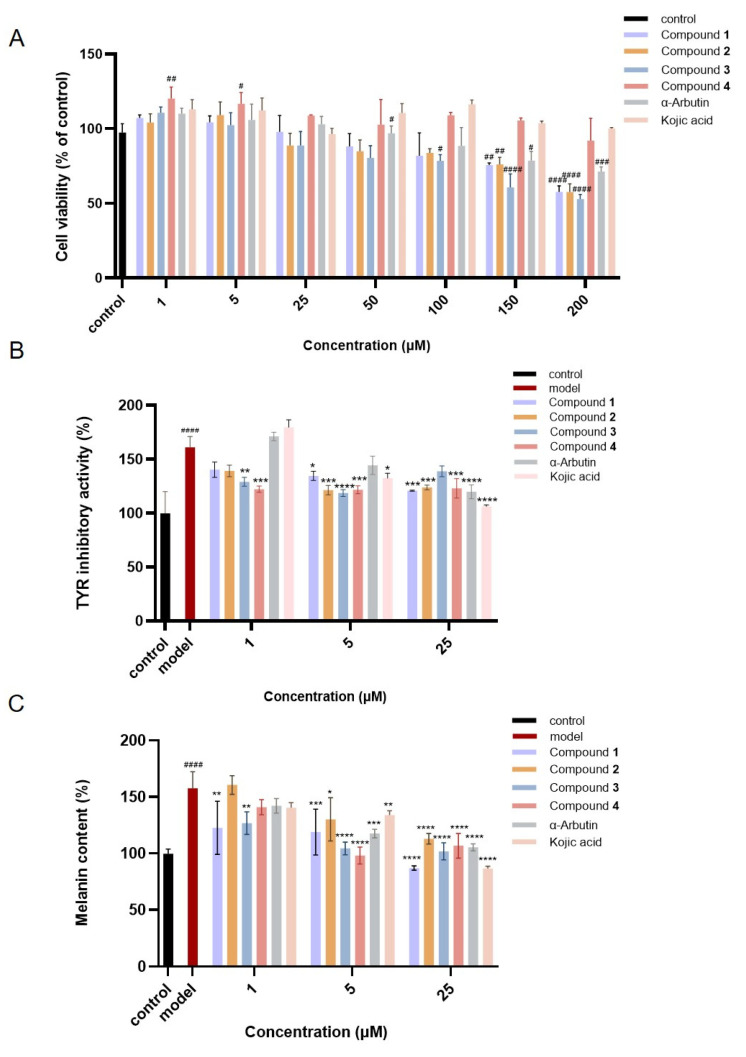
Effect of compounds from *A. sparsifolia* on B16 melanoma cells: (**A**) cell cytotoxicity; (**B**) tyrosinase inhibitory activity; (**C**) melanin content reduction ability. α−Arbutin and kojic acid serve as positive controls. (^#^ *p* < 0.05, ^##^ *p* < 0.01, ^###^ *p* < 0.001 and ^####^ *p* < 0.0001, compared with the control group; * *p* < 0.05, ** *p* < 0.01, *** *p* < 0.001 and **** *p* < 0.0001, compared with the model group).

Initially, the effect of active compounds **1**–**4** from *A. sparsifolia* at concentrations ranging from 1 to 200 μM on the viability of B16 melanoma cells was tested. As shown in Figure 7A, the safe concentration range for these compounds in B16 melanoma cells was determined to be between 1 and 25 μM. All the compounds except **4** showed an obvious toxic effect at 50–200 μM. In contrast, compound **4** and the positive control kojic acid were safe enough even at 200 μM. Subsequently, the inhibitory effect of these compounds on tyrosinase activity in B16 cells was evaluated. The intracellular tyrosinase activity in the α-MSH-induced B16 cells increased to 160.91 ± 10.00% of the normal cells, while all four of the compounds could significantly reduce tyrosinase activity at concentrations of 1, 5, and 25 μM. Compound **4** (1 μM) displayed the most significant inhibitory effect by reducing it to 122.13 ± 2.96% of the normal cells. At a concentration of 5 μM, the inhibitory effect of all the compounds surpassed the two positive controls, α-arbutin and kojic acid. Similarly, all the compounds at the three concentrations significantly inhibited the production of melanin in the model cells, among which compounds **3** and **4** (5 μM) exhibited the best inhibition by almost recovering the melanin level to the normal values (104.35 ± 5.65% and 98.04 ± 7.48% of the normal cells, respectively), which was much better than the two positive controls.

**Figure 8:**
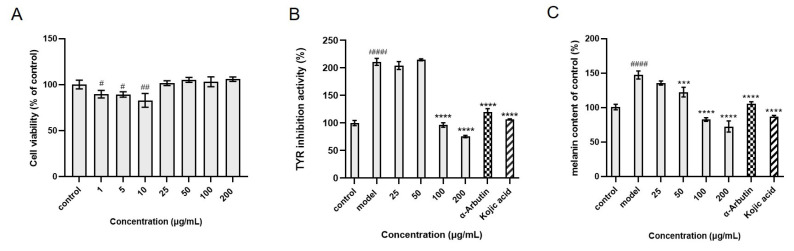
Effects of the active fraction from *C. arabica* (S5) on B16 melanoma cells: (**A**) cell cytotoxicity of compounds on B16 melanoma cells, (**B**) tyrosinase inhibitory activity, (**C**) melanin content reduction ability. α−Arbutin and kojic acid serve (25 μM) as positive controls. (^#^ *p* < 0.05, ^##^ *p* < 0.01 and ^####^ *p* < 0.0001, compared with the control group; *** *p* < 0.001 and **** *p* < 0.0001, compared with the model group).

Since the active fraction (S5) fished out from *C. arabica* was mainly composed of fatty acids, which were difficult to isolate, we directly tested its inhibitory effects on tyrosinase activity and melanin production in the α-MSH-induced B16 melanoma cells. As shown in Figure 8A, it was surprising that S5 showed a degree of toxicity at low concentrations of 1–10 μg/mL, while it was safe at 25–200 μg/mL. Interestingly, at a high concentration of 100 μg/mL, S5 entirely recovered the intracellular tyrosinase activity and decreased the melanin production to a level lower than the control, while at 200 μg/mL it exhibited much stronger inhibition on both levels.

## 3. Materials and Methods

### 3.1. Materials and Chemicals

The B16 melanoma cell lines were sourced from the Shanghai Cell Bank, Chinese Academy of Sciences (Shanghai, China). The CD04, Dulbecco’s modified Eagle’s medium (DMEM), penicillin-streptomycin, fetal bovine serum (FBS), and the other culture reagents were supplied by Gibco (Carlsbad, CA, USA). Cell Counting Kit-8 (CCK-8) was purchased from the ProteinTech Group (Chicago, IL, USA). The α-MSH was bought from Beyotime Biotechnology Co., Ltd. (Shanghai, China). HPLC-grade methanol and acetonitrile were provided by JT Technology (Beijing, China). *A. sparsifolia* and *C. arabica* were purchased from a herbal market in Chengdu of Sichuan Province of the People’s Republic of China, and the voucher specimens (2021-02 for the former and 2021-03 for the latter) were deposited in the Herbarium of Chengdu Institute of Biology, Chinese Academy of Sciences. The pcDNA3.1 (+) plasmid was synthesized by GenScript Corporation (GenScript, Nanjing, China).

The Shimadzu LC-30AD UFLC system was equipped with an SPD-20A UV detector and a BEH C18 column (2.1 × 100 mm, 1.7 μm, Waters, Manchester, UK), and QTRAP5500-MS (AB Sciex, Redwood City, CA, USA) was used for HPLC-MS analysis. Analytical HPLC was performed on a Shimadzu LC-20AD HPLC system equipped with an SPD-20A UV detector and a reverse-phase Kromasil 100-5-C18 column (4.6 × 250 mm, 5 μm, AkzoNoble, Bohus, Sweden). Semi-preparative HPLC was conducted on the same Shimadzu LC-20AD system but with a COSMOSIL C18 column (10 × 250 mm, 5 μm, Nacalai Tesque Corporation, Kyoto, Japan). The UV absorbance was measured by a MULTISKAN GO spectrophotometric plate reader (Thermo Scientific, Waltham, MA, USA).

### 3.2. Cell Culture and CHO@hTYR Construction

The CHO-S (Invitrogen, Carlsbad, CA, USA) cells were cultured in CD04 medium at 37 °C in 5% CO_2_. The pCDNA3.1 (+) vector was used to express human tyrosinase. Genes encoding hTYR-CD8hinge-CD8TM-CD8cyto-EGFP were synthesized, then cloned into the pCDNA3.1 (+) expression vector via HindIII/NotI double enzyme digestion, resulting in plasmid pCDNA3.1/hTYR. The plasmid was transferred into CHO-S cells using the electroporation method, and positive cells were obtained through G418 pressure screening. Among them, CD8TM serves as the transporter of hTYR to the cell surface, while the CD8hinge functions as a “stalk” that anchors hTYR on the cell surface [34,35]. The ositive cells of CHO-S-hTYR express human tyrosinase in the form of membrane proteins on the CHO-S cell. The EGFP tag was used to display the correctness of expression on the membrane. In the meantime, the empty vector of pCDNA3.1 (+) was transfected into CHO-S cells by the same method to be used as blank control in the following steps.

### 3.3. Fluorescence Assay of CHO@hTYR

The efficiency of CHO@hTYR was directly evaluated by the fluorescence. The fluorescence intensity of CHO@hTYR at wavelength 507 nm was recorded under the excitation wavelength of 488 nm. The CHO cells transfected with an empty vector were used as a blank control.

### 3.4. Enzymatic Activity and Stability of CHO@hTYR

The tyrosinase activity of CHO@hTYR was verified following a previous method [36,37]. The CHO@hTYR cells (1 × 10^4^) were centrifuged at 1000 rpm for 5 m and washed with PBS; then, 5 mM L-DOPA was added to be incubated with the surfaced recombinant cells for 30 m at 37 °C, after which the generation amount of dopaquinone was reflected by the absorbance value at 405 nm. Meanwhile, the stability of the CHO@hTYR cells was assessed by the method above. The CHO@hTYR cells were stored at 4 °C, and the same number of recombinant cells were taken to detect the relative tyrosinase activity every 24 h. Additionally, the activity of the CHO@hTYR stored for different numbers of days was detected to comprehensively evaluate the stability of the CHO@hTYR.

### 3.5. Optimization of Ligand Fishing Procedure

To optimize the ligand fishing procedure utilizing CHO@hTYR, several parameters were evaluated, including the number of CHO cells, the incubation time, the incubation buffer, the desorption time, and the desorption solvent. The tyrosinase activity on the cell surface following the fishing process served as the evaluation index for optimizing the fishing program. Under the optimal fishing conditions, the reusability of CHO@hTYR was investigated, and quercitrin selected as the target compound for this procedure. Additionally, the lowest detection limit of recombinant cells for fishing was assessed, that is, the concentration of quercitrin ligand at a standard deviation-to-slope ratio of 3, which was the concentration of quercitrin.

### 3.6. Selectivity Validation of Ligand Fishing with CHO@hTYR

To verify whether CHO@hTYR could specifically adsorb to the tyrosinase inhibitors, a mixture of tyrosinase-positive and tyrosinase-negative compounds was prepared to assess the specific adsorption capacity of recombinant cells. The experimental procedure involved incubating 5 × 10^4^ cells with 1 mL of the mixed solution (configured to 0.1 mg/mL) at 37 °C for 30 min. Following incubation, the mixture was centrifuged and washed three times with Tris-HCL. For the final wash, 30% methanol was used as the desorption solvent to extract the potential ligands designated as mS5. The obtained solutions, mS0 and mS5, were subsequently analyzed using HPLC, with CHO cells serving as blank controls in the experiments.

### 3.7. Ligand Fishing from A. sparsifolia and C. arabica

*A. sparsifolia* and *C. arabica* were ground into a fine powder and then soaked in 80% ethanol at a material-to-liquid ratio of 1:5. The resultant extract was evaporated, concentrated, and stored at −20 °C after three extraction cycles. The obtained *A. sparsifolia* and *C. arabica* extract was prepared as a 1 mg/mL solution designated as S0. Subsequently, a 1 mL S0 solution was mixed with an amount of CHO@hTYR cells at 37 °C for 30 min to facilitate the interaction between the HTyr and the potential ligand compounds. After incubation, the mixture was centrifuged at 1000 rpm for 5 min, and the supernatant was discarded. The cells were then resuspended in Tris-HCL and gently shaken three times to eliminate false positive ligands. Finally, a 30% methanol solution was employed to desorb the ligand compounds from the surface of the hTYR and was named S5. The CHO cells, which did not express human tyrosinase, were subjected to the same fishing procedure as the blank control. The final desorption solvent was labeled as S5-blank. S0, S5, and S5-blank were analyzed using HPLC under the following conditions: UV detection wavelength of 220 nm, column temperature of 35 °C, injection volume of 20 μL, mobile phase consisting of solvent A (0.1% formic acid/water) and solvent B (methanol), gradient elution of 30–100% B over 0–35 min, and a flow rate of 0.8 mL/min.

### 3.8. Isolation and Identification of Tyrosinase Ligands

The methanol extract of *A. sparsifolia* (83 g) was applied to a silica gel column (100–200 mesh) eluted by dichloromethane–methanol (from 50:1 to 0:1) to afford eight fractions. Compounds **1** (7.7 mg) and **3** (27.0 mg) were isolated from Fr. 3 and Fr. 7, respectively. Compounds **2** (5.4 mg) and **4** (5.6 mg) were purified by semi-preparative HPLC (60% MeOH) from Fr. 1 and Fr. 7, respectively. The purified compounds were subjected to MS and NMR analyses. Electrospray ionization MS analysis was conducted in positive ion mode, at a scan range of 50–1000 *m*/*z*, a spray voltage of 3.00 kV, a source temperature of 120 °C, a desolvation temperature of 450 °C, a cone gas flow rate of 50 L/h, and a desolvation gas flow rate of 800 L/h. NMR data were recorded by a Bruker DRX-600 spectrometer (Bruker, Rheinstetten, Germany).

The methanal extract of *C. arabica* (9 g) was fractionated with petroleum ether, which was then subjected to silica gel column chromatography (100–200 mesh), eluted with petroleum ether-ethyl acetate (200:1 to 50:1) to obtain the active fraction (3.5 g), as indexed by the ligand fishing. The fatty acids in this fraction were analyzed by GC-MS (Agilent 5977B, Agilent, Waldbronn, Germany) equipped with an HP-5ms chromatographic column (30 m × 0.25 mm × 0.25 µm). The chromatographic conditions were as follows: an initial temperature of 40 °C, held for 5 min, followed by a ramp of 10 °C/min to 250 °C, and a subsequent increase of 5 °C/min to 280 °C, which was maintained for an additional 5 min. The mass spectrometry parameters included an electron impact source with the temperature of 230 °C, an interface temperature of 250 °C, an electron energy of 65 eV, and a mass scan range of 30–550 amu.

### 3.9. Tyrosinase Inhibitory Activity in B16 Melanoma Cells

#### 3.9.1. Cell Culture and Cytotoxicity Test

The B16 melanoma cells were maintained in fresh DMEM medium supplemented with 10% FBS and 1% penicillin-streptomycin at 37 °C under 5% CO_2_. The cells were cultured at regular intervals to ensure logarithmic growth. The cytotoxicity of the active fraction against B16 melanoma cells was assessed through the CCK8 assay. Upon reaching 80–90% confluence, the B16 melanoma cells were detached with trypsin and seeded in 96-well plates at a density of 1.0 × 10^4^ cells/well for 24 h. Subsequently, 100 μL of medium containing various concentrations of the test sample (active fraction and the positive control) ranging from 1 to 200 μM was cultured for an additional 24 h. Following this, 10 μL of CCK8 reagent was added to each well and reacted at 37 °C for 1 h. Absorbance was measured using a microplate reader at a wavelength of 450 nm. The cytotoxicity was expressed as the percentage of cell viability compared with the blank control.

#### 3.9.2. Tyrosinase Activity Assay

The B16 melanoma cells (2 × 10^5^ cells/well in a 6-well plate) were exposed to various concentrations of the test sample in a 1 μΜ α-MSH medium for 48 h at 37 °C. The cells were then lysed using 1% Triton X-100 and sonication, followed by the addition of levodopa (5 mM) to each well for further incubation at 37 °C for 30 min. The absorbance value was then measured at 475 nm. The relative activity was displayed according to the ratio of the OD value of the sample to the blank control.

#### 3.9.3. Melanin Content Assay

The B16 melanoma cells were incubated at a density of 1.25 × 10^5^ cells/well in 6-well plates for 24 h. Next, the active fractions of different concentrations in 1 μM α-MSH medium were added and cultured for another 72 h before the cells were washed with ice-cold PBS and lysed by trypsinization. The synthesis of melanin was disrupted by using 1 M NaOH with 10% DMSO heating at 80 °C for 60 min. Finally, the absorbance of each sample was measured at 405 nm, and the melanin content was expressed as a percentage relative to the blank control group.

### 3.10. Statistical Analysis

All the experiments tested were replicated at least three times. The data were expressed as mean ± SD and were analyzed using GraphPad Prism, version 10.0.2 software.

## 4. Conclusions

In this study, a CHO cell line stably expressing the human tyrosinase gene (CHO@hTYR) was constructed. The recombinant cell exhibited excellent storage stability. The ligand fishing strategies were established utilizing the recombinant cell to screen compounds from complex natural product systems efficiently and accurately. The method was validated as sensitive, time-efficient, and specific. This fishing technique was employed to identify tyrosinase inhibitors from *A. sparsifolia* and *C. arabica*. Notably, four effective tyrosinase inhibitors, 4-methoxy-5-methyl coumarin (**1**), cupressuflavone (**2**), amentoflavone (**3**), and 3,4-dimethoxy-5-methyl coumarin (**4**), were isolated from *A. sparsifolia* for the first time. The active fractions in *C. arabica* were identified as linolenic acid, palmitic acid, ricinoleic acid, and palmitic acid. According to the B16 intracellular activity tests, the active component (S5) from *C. arabica* and the four compounds from *A. sparsifolia* significantly inhibited intracellular tyrosinase activity and melanin production. This work supplied a novel approach to ligand fishing, offering new avenues for the rapid screening of novel tyrosinase inhibitors. Furthermore, the scientific foundation was laid for *A. sparsifolia* and *C. arabica* as natural inhibitors of melanin production.

## Data Availability

The raw data supporting the conclusions of this article will be made available by the authors on request.

## Supplementary Materials

The following supporting information can be downloaded at: www.mdpi.com/article/10.3390/molecules30010030/s1, Table S1: The Primer list in vector construction, Figure S1: Agarose gel electrophoresis of colony PCR, Figure S2: Gene sequencing result of No.13 strain, Figure S3: Calibration curve of mushroom tyrosinase activity, Figure S4: Calibration curves, Figure S5: Reusability of the CHO@hTYR, Figure S6: Mass spectra of 4-methoxy-5-methyl coumarin (A), cupressuflavone (B), amentoflavone (C), and 3, 4-dimethoxy-5-methyl coumarin (D), Figure S7: NMR spectra of 4-methoxy-5-methyl coumarin (A), cupressuflavone (B), amentoflavone (C), and 3, 4-dimethoxy-5-methyl coumarin (D), Table S2: ^1^H and ^13^C NMR data for compounds **1**–**4** in CD_3_OD, Figure S8: GC-MS spectra of the active fraction from *C. arabica* (A) and the standard mixture (B), Figure S9: HPLC chromatograms of ligand fishing using MNP@mTYR from *A. sparsifolia*, and chemical structures of quercitrin and hypolaetin, Figure S10: HPLC chromatograms of ligand fishing using MNP@mTYR from *C. arabica*, Figure S11: Effects of compounds from *A. sparsifolia* on B16 melanoma cells.
