# Supplementary material for: Human Tyrosinase Displayed on the Surface of Chinese Hamster Ovary Cells for Ligand Fishing of Tyrosinase Inhibitors from Medicinal Plants
Source: Molecules. 2024 Dec 25;30(1):30. doi: 10.3390/molecules30010030 (PMC11721096; doi:10.3390/molecules30010030)
Supplement: Supplementary file 1 [file molecules-30-00030-s001.zip › molecules-3353701-supplementary.pdf]

Supplementary Data

Part S1. Construction of pCDNA3.1/hTYR

The *homo tyrosinase* primer sequence (Table 1) was synthesized by Sangon Biotech Co., Ltd., (Shanghai). As illustrated in Figure S1, the results of the colony PCR identification indicated that No. 3 and No. 13 bacterial colonies were in line with expectations. The gene sequencing result of No. 13 strain was correct (Fig. S2).

Table S1. The Primer list in vector construction

Primers	Vector
F-CACGCCGCTAGACCCATGCTGCTGGCCGTG<br>R-GGCGGGGGTTGTTGTCAGGTGAGACTGGTA	pcDNA3.1 (+)

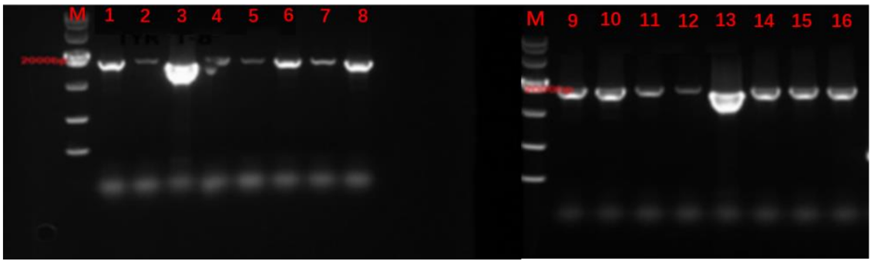

Figure S1. Agarose gel electrophoresis of colony PCR: slots 1 and 10: marker.

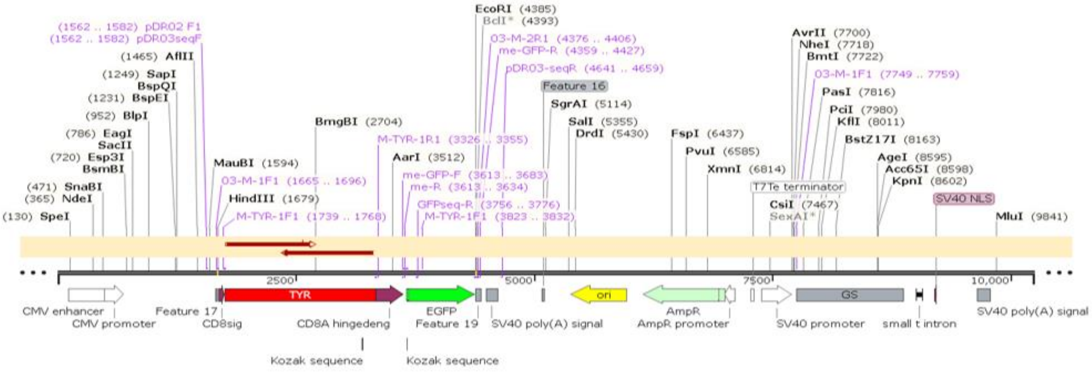

Figure S2. Gene sequencing result of No.13 strain.

Part S2. The enzymatic activity of CHO@hTYR

The standard curve (Figure S3) with the concentration of mushroom tyrosinase plotted on the x axis and the OD values at 475 nm, representing the amount of reaction product on the y axis. The product quantity generated by the catalytic substrate of 1×10^4 recombinant cells was then substituted into the standard curve to reflect the corresponding enzyme activity of mushroom tyrosinase.

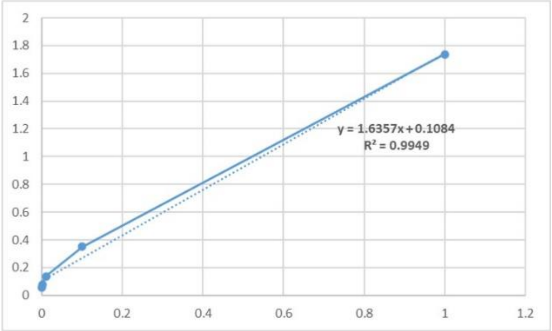

Figure S3. Calibration curve of mushroom tyrosinase activity.

Part S3. The limit of detection (LOD) for ligand fishing

Quercitrin was prepared to a series concentration of solutions (0.1, 1, 10, 100, 1000 μM). A total of 5 × 10^4 CHO@hTYR cells were utilized to perform the ligand fishing with those solutions (S0) as outlined in Section 3.6 of the text. The lowest detection (LOD) limit of the method was determined by the corresponding concentration of quercitrin when the peak area value of S5 reached 3 times of the noise value.

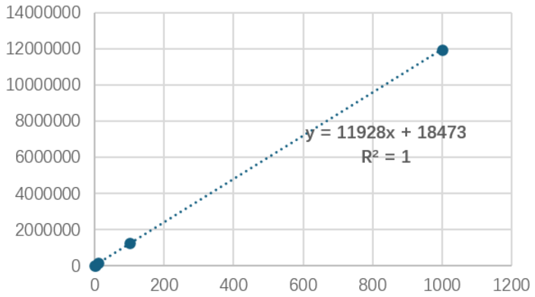

Figure S4. Calibration curve.

Part S4. Reusability of the recombinant cells

A total of 5 × 10^4 of CHO@hTYR cells was repeatedly used for ligand fishing with 1 mL of quercitrin (100 μM) as outlined in Section 3.6 of the text. As shown in Figure S5, the peak area of quercitrin after sixth fishing is nearly indistinguishable from the noise. Consequently, it can be concluded that the recombinant cells can be reused for five rounds.

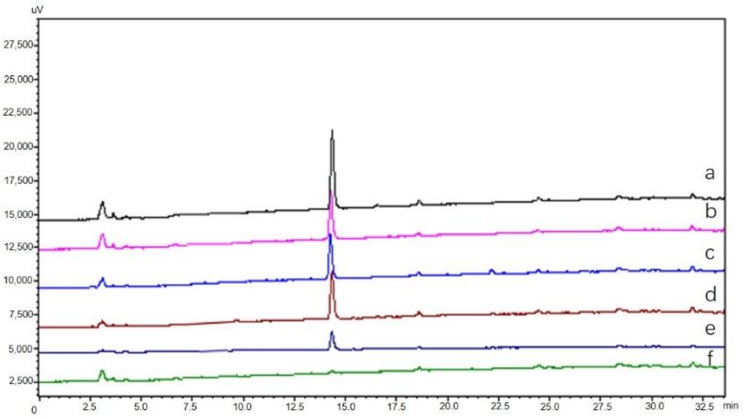

Figure S5. Reusability of the CHO@hTYR: a-f refer to 1st-6th time of ligand fishing.

Part S5. Structure identification of hTYR inhibitors from *A. sparsifolia.* and *C. arabica.*

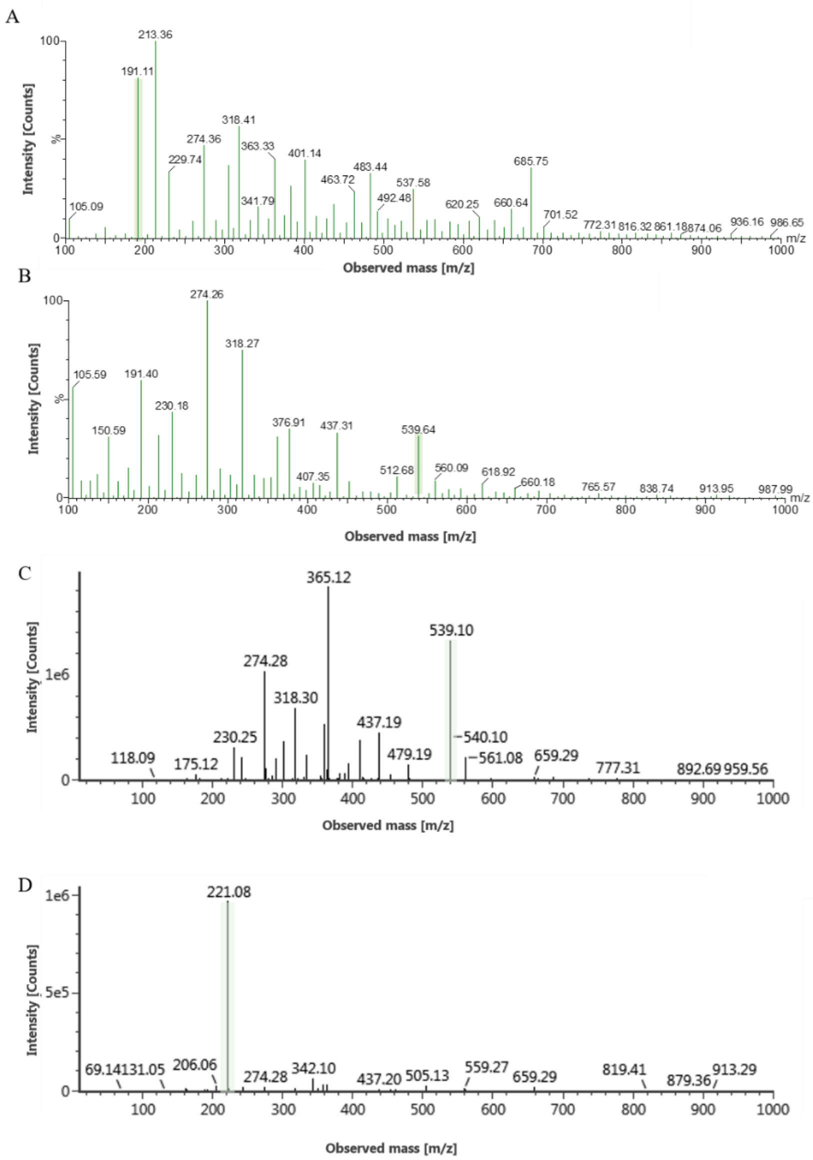

Figure S6. Mass spectra of 4-methoxy-5-methyl coumarin (A), cupressuflavone (B), amentoflavone (C), and 3, 4-dimethoxy-5-methyl coumarin (D).

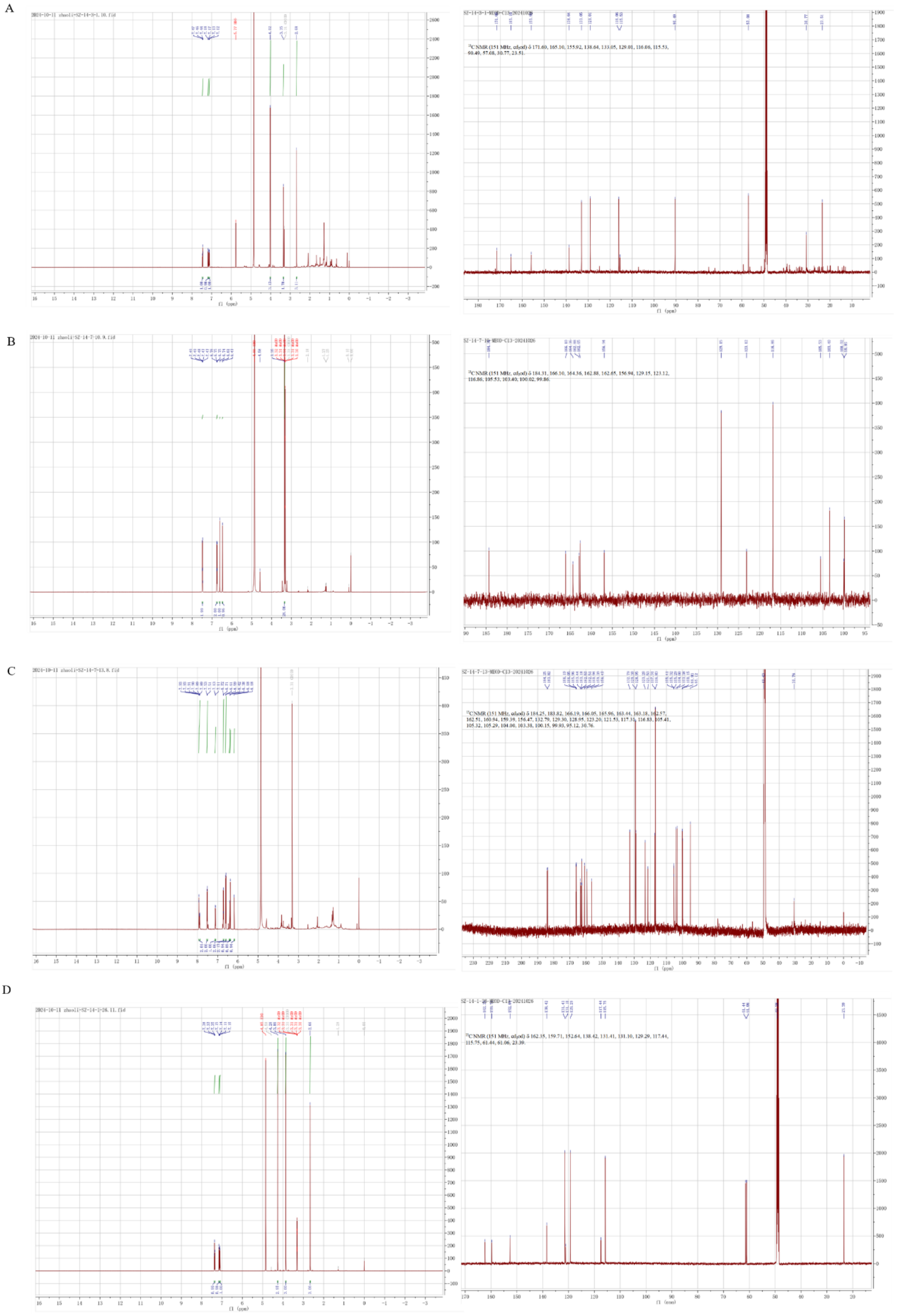

Figure S7. NMR spectra of 4-methoxy-5-methyl coumarin (A), cupressuflavone (B), amentoflavone (C), and 3, 4-dimethoxy-5-methyl coumarin (D). Left: ^{1}H NMR (600 MHz, CD_3OD) spectra, right: ^{13}C NMR (150 MHz, CD_3OD) spectra.

Table S2: ^{1}H and ^{13}C NMR data for compounds **1−4** in CD_3OD

No.	Compound **1**		Compound **2**		Compound **3**		Compound **4**	
	δ_H	δ_C, type	δ_H	δ_C, type	δ_H	δ_C, type	δ_H	δ_C, type
2		171.6, C		164.4, C		163.2, C		162.4, C
3	5.77 (s)	90.5, CH	6.61 (s)	103.4, CH	6.41 (s)	103.8, CH	4.26 (s)	61.1, OMe
4		165.1, C		184.3, C		184.3, C		159.7, C
5		138.6, C		162.7, C		166.1, C		138.4, C
6	7.18 (d, 8.3)	129.0, CH	6.47 (s)	99.9, CH	6.18 (d, 2.1)	99.9, CH	7.14 (d, 8.3)	131.1, CH
7	7.45 (t, 7.8)	133.1, CH		166.1, C		156.5, C	7.37 (t, 7.9)	131.4, CH
8	7.12 (d, 7.5)	116.1, CH		100.0, C	6.72 (d, 3.1)	95.1, CH	7.10 (d, 7.5)	129.3, CH
9		155.9, C		156.9, C		159.4, C		152.6, C
10		115.5, C		105.5, C		105.3, C		117.4, C
11	3.34 (s)	57.1, OMe					3.86 (s)	61.4, CH_3
12	2.67 (s)	23.5, CH_3					2.66 (s)	23.4, OMe
1’				123.1, C		123.2, C		
2’			7.48 (d, 8.76)	129.2, CH	7.93 (d, 6.0)	132.8, CH		
3’			6.75 (d, 8.83)	116.9, CH		121.5, C		
4’				162.9, C		163.4, C		
5’			6.75 (d, 8.83)	116.9, CH	7.12 (d, 8.7)	121.5, CH		
6’			7.48 (d, 8.76)	129.2, CH	7.89 (dd, 2.5, 8.6)	129.0, CH		
7’				166.1, C				
8’				100.0, C				
1”								
2”				164.4, C		166.0, C		
3”			6.61 (s)	103.4, CH	6.47 (s)	104.0, CH		
4”				184.3, C		183.8, C		
5”				162.7, C		166.2, C		
6”			6.47 (s)	99.9, CH	6.37 (s)	100.2, CH		
7”						162.6, C		
8”						105.3, C		
9”				156.9, C		160.9, C		
10”				105.5, C		105.4, C		
1”’				123.1, C		123.0, C		
2”’			7.48 (d, 8.76)	129.2, CH	6.60 (d, 8.8)	129.2, CH		
3”’			6.75 (d, 8.83)	116.9, CH	6.60 (d, 8.8)	116.8, CH		
4”’				162.9, C		162.5, C		
5”’			6.75 (d, 8.83)	116.9, CH	6.60 (d, 8.8)	116.8, CH		
6”’			7.48 (d, 8.76)	129.2, CH	7.52 (d, 8.8)	129.3, CH		

Compounds **1−4** were measured at ^{1}H NMR (600 MHz) and ^{13}C NMR (150 MHz), and δ in ppm and J in Hz.

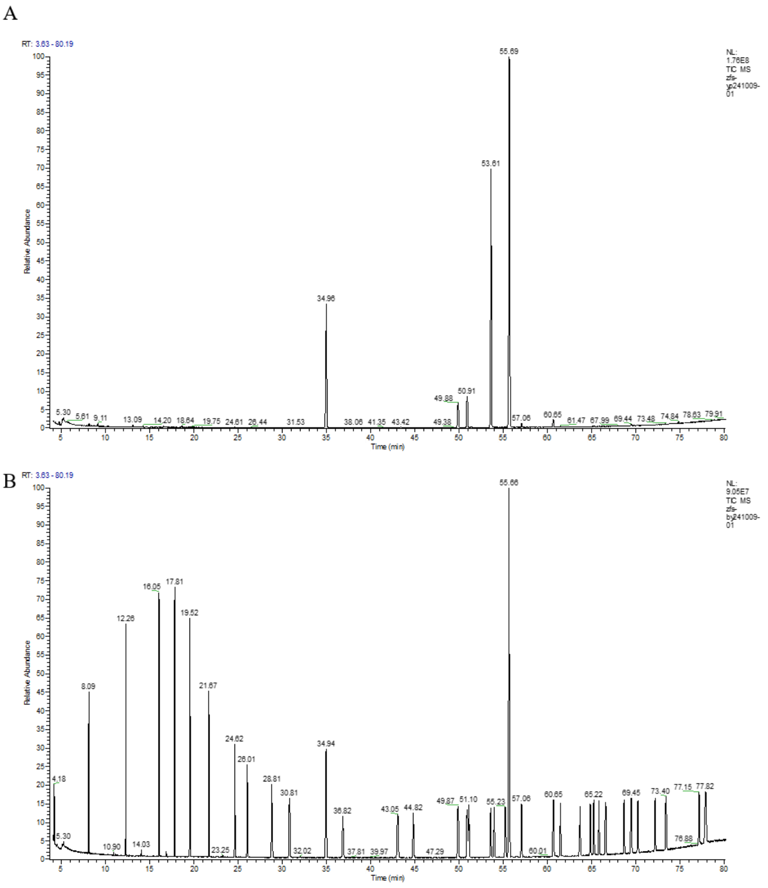

Figure S8. GC-MS spectra of the active fraction from *C. arabica* (A), and the standard mixture (B).

Part S6. Ligand fishing of tyrosinase ligands from *A. sparsifolia* and *C. arabica* using MNP@mTYR

Two compounds were fished out from *A. sparsifolia* using MNP@mTYR (Figure S9) which were identified as quercitrin and hypolaetin. For *C. Arabica*, it is noteworthy that MNP@mTYR could not catch any compounds (Figure S10). The above results differ significantly from those obtained using CHO@hTYR. Additionally, we evaluated the effects of quercitrin and hypolaetin on intracellular tyrosinase activity and melanin content in B16 melanoma cells. As shown in Figure S11, both compounds (MNP@mTYR) significantly inhibited the activity of tyrosinase in B16 melanoma cells, however, they were less effective than the four compounds fished out by CHO@hTYR in reducing the intracellular melanin content.

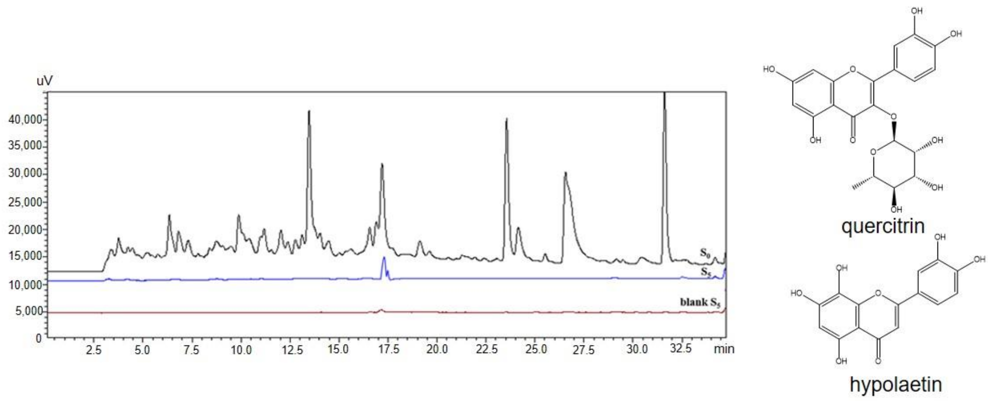

Figure S9. HPLC chromatograms of ligand fishing using MNP@mTYR from *A. sparsifolia*, and chemical structures of quercitrin and hypolaetin.

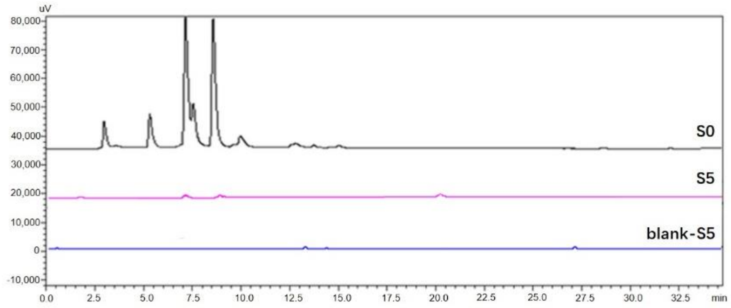

Figure S10. HPLC chromatograms of ligand fishing using MNP@mTYR from *C. arabica*.

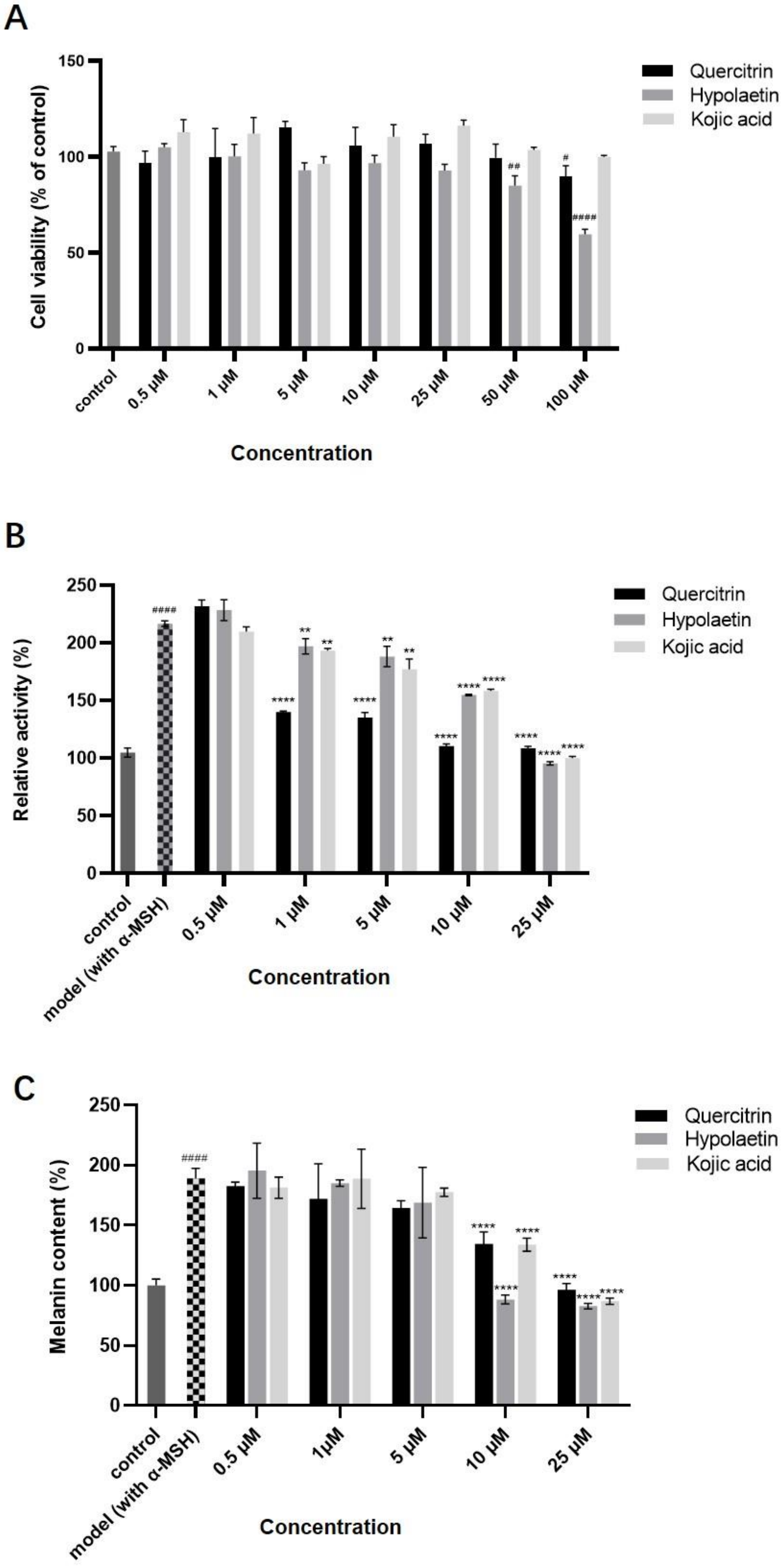

Figure S11. Effects of compounds from *A. sparsifolia* on B16 melanoma cells. (A) Cell cytotoxicity; (B) tyrosinase inhibitory activity; (C) melanin content reduction ability. Kojic acid serves as positive control. ($^{\#}p < 0.05$, $^{\#\#}p < 0.01$, and $^{\#\#\#\#}p < 0.0001$, compared with the control

group; **$p < 0.01$, and ****$p < 0.0001$, compared with the model group).
